# Validating the Gap-Startle Paradigm for Tinnitus Detection: A Machine Learning Approach in CBA/CaJ Mice

**DOI:** 10.64898/2026.09.05.747694

**Published:** 2026-09-09

**Authors:** Alyssa B. Price, Dimitri L. Brunelle, Collin R. Park, Andrea S. Lowe, Edward Lobarinas, Joseph P. Walton

## Abstract

Tinnitus is one of the most common hearing disorders affecting one-third of Americans and is defined as the buzzing or ringing sound one perceives in one or both ears in the absence of an acoustic stimulus. One objective method for tinnitus screening in rodents is gap prepulse inhibition of the acoustic startle reflex (GPIAS), a reduction in the abrupt motor response elicited by an intense auditory stimulus following a silent gap in noise. Reduced inhibition by gaps embedded in narrow-band noise is hypothesized to reflect the primary tinnitus pitch, as the tinnitus “fills-in” the gap. However, Lobarinas et al. (2013) identified a critical limitation: after acoustic trauma or hearing loss, rodents often exhibit markedly diminished acoustic startle reflexes, creating a “floor effect” where further suppression becomes undetectable even if gap perception remains intact, leading to false-positive tinnitus screening results. To address this limitation, we utilized the CBA/CaJ mouse model to assess tactile airpuff modification efficacy and employed a machine learning algorithm to classify startle responses, achieving 98% accuracy in differentiating startles from non-startles. We induced unilateral conductive hearing loss via ear plugging and found enhanced gap detection ability, contrasting with false-positive tinnitus indicators observed in rats by Lobarinas and colleagues. We also pharmacologically induced tinnitus via sodium salicylate, revealing frequency-specific alterations in gap detection patterns. Our findings suggest that differences in data analysis methodology, specifically using a machine learning algorithm to filter non-startle responses, may explain species-specific differences between mouse and rat models and significantly improve GPIAS validity as a tinnitus screening assessment tool.

**Highlights:**

• Gap-startle paradigm objectively screens for tinnitus in rodent models.

• Machine learning classification achieved 98% accuracy in startle detection.

• Enhanced analysis resolved floor effects that previously limited paradigm validity.

• Species differences explained by improved data filtering methodology.

## Full Text Availability

The license terms selected by the author(s) for this preprint version do not permit archiving in PMC. The full text is available from the preprint server.

